# Acute kidney injury according to RIFLE criteria in an ICU: incidence and mortality impact

**DOI:** 10.1186/cc12646

**Published:** 2013-06-19

**Authors:** AR Santana, FF Amorim, FB Soares, LG de Souza Godoy, L de Jesus Almeida, TA Rodrigues, G Menezes de Andrade Filho, TA Silva, JL de Souza, KCM Ogliari, PN Ferreira, APP Amorim, EB de Moura, JA de Araújo Neto, M de Oliveira Maia

**Affiliations:** 1 Unidade de Terapia Intensiva Adulto do Hospital Santa Luzia, Asa Sul, Brasília, DF, Brazil

## Introduction

Acute kidney injury (AKI) is a very common condition in hospitalized patients, especially in ICUs. It is also closely related to adverse patient outcomes, mortality rates as high as three-quarters and as many as 13% need of renal support after hospital discharge. A systematic review demonstrated a close correlation between AKI according to the RIFLE criteria and mortality. The objective of this study was to evaluate the incidence of AKI according to the RIFLE criteria and the impact of each category on mortality in an ICU.

## Methods

A retrospective cohort study was conducted with patients admitted to the adult ICU of Hospital Santa Luzia, Brasilia, DF, Brazil, in the period of 6 months. Patients were categorized as Risk (R), Injury (I), Failure (F), or without AKI according to RIFLE criteria. Patients with a previous diagnosis of chronic kidney disease were excluded.

## Results

**Figure 1:**
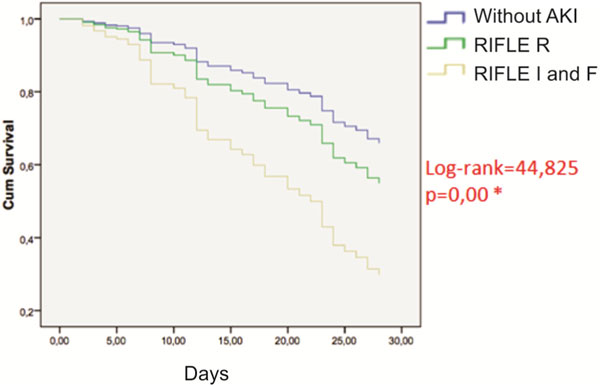


A total of 626 patients were included. Average age was 60 ± 20 years, 326 were male (50.8%), APACHE II was 9 ± 6, 67.1% (*n = *326) were nonsurgical, and the mortality rate was 12.3% (*n = *77). According to RIFLE criteria, 148 had AKI. Eighty-three patients were classified as R (13.3%, mortality rate of 21.7%), 43 as I (6.9%, mortality rate of 53.5%), and 22 patients as F (3.5%, mortality rate of 54.5%). The relative risk (RR) of death in patients classified as R was 2.72 (95% CI: 1.26 to 4.09), I was 11.27 (95% CI: 5.81 to 21.83), and F was 9.91 (95% CI: 4.14 to 23.94). Analyzing all patients with AKI, the RR of death was 11.22 (95% CI: 6.57 to 19.17). Eight (1.3%) patients underwent renal replacement therapy during ICU hospitalization, and mortality in these patients was 75% (RR: 23.11, 95% CI 4.58 to 116.71). Significant difference was observed in the Kaplan-Meier survival curves of the patients with or without AKI at 28 days (*P *= 0.00). See Figure 1.

## Conclusion

AKI according to RIFLE criteria was associated with an increased mortality for all categories, mainly in patients with criteria to injury and acute kidney failure, and notably those who needed renal replacement therapy.
